# Fat infiltration of the posterior paraspinal muscles is inversely associated with the fat infiltration of the psoas muscle: a potential compensatory mechanism in the lumbar spine

**DOI:** 10.1186/s12891-023-06967-w

**Published:** 2023-10-27

**Authors:** Maximilian Muellner, Henryk Haffer, Erika Chiapparelli, Yusuke Dodo, Jennifer Shue, Ek T. Tan, Jiaqi Zhu, Matthias Pumberger, Andrew A. Sama, Frank P. Cammisa, Federico P. Girardi, Alexander P. Hughes

**Affiliations:** 1 Spine Care Institute, Hospital for Special Surgery, New York City, NY USA; 2 Center for Musculoskeletal Surgery, Charité - Universitätsmedizin Berlin, corporate member of Freie Universität Berlin, Humboldt-Universität Zu Berlin, Berlin, Germany; 3 Department of Radiology and Imaging, Hospital for Special Surgery, Weill Cornell Medicine, New York City, NY USA; 4 Biostatistics Core, Hospital for Special Surgery, New York City, NY USA

**Keywords:** Connective tissue, Muscle quality, Spinal fusion, Spine, Lumbar lordosis, Spinal stability, Posterior paraspinal muscles, Psoas muscle

## Abstract

**Background:**

The function of the paraspinal muscles and especially the psoas muscle in maintaining an upright posture is not fully understood. While usually considered solely as a hip flexor, the psoas muscle and its complex anatomy suggest that the muscle has other functions involved in stabilizing the lumbar spine. The aim of this study is to determine how the psoas muscle and the posterior paraspinal muscles (PPM; *erector spinae and multifidus*) interact with each other.

**Methods:**

A retrospective review including patients undergoing posterior lumbar fusion surgery between 2014 and 2021 at a tertiary care center was conducted. Patients with a preoperative lumbar magnetic resonance imaging (MRI) scan performed within 12 months prior to surgery were considered eligible. Exclusion criteria included previous spinal surgery at any level, lumbar scoliosis with a Cobb Angle > 20° and patients with incompatible MRIs. MRI-based quantitative assessments of the cross-sectional area (CSA), the functional cross-sectional area (fCSA) and the fat area (FAT) at L4 was conducted. The degree of fat infiltration (FI) was further calculated. FI thresholds for FI_PPM_ were defined according to literature and patients were divided into two groups (< or ≥ 50% FI_PPM_).

**Results:**

One hundred ninetypatients (57.9% female) with a median age of 64.7 years and median BMI of 28.3 kg/m^2^ met the inclusion criteria and were analyzed. Patients with a FI_PPM_ ≥ 50% had a significantly lower FI in the psoas muscle in both sexes. Furthermore, a significant inverse correlation was evident between FI_PPM_ and FI_Psoas_ for both sexes. A significant positive correlation between FAT_PPM_ and fCSA_Psoas_ was also found for both sexes. No significant differences were found for both sexes in both FI_PPM_ groups.

**Conclusion:**

As the FI_PPM_ increases, the FI_Psoas_ decreases. Increased FI is a surrogate marker for a decrease in muscular strength. Since the psoas and the PPM both segmentally stabilize the lumbar spine, these results may be indicative of a potential compensatory mechanism. Due to the weakened PPM, the psoas may compensate for a loss in strength in order to stabilize the spine segmentally.

## Background

Upright human posture and bipedalism are distinguishing characteristics of humans from other primates [1–3]. The standing stance is balanced, consumes little energy, and can be maintained for a long time. A number of factors contribute to the standing posture, including the spine that connects the upper extremities to the lower extremities and enables a stable, low-energy upright posture [3].

The stabilization of the spine is based on several factors such as an active and passive stabilization system in addition to a neural control system [4, 5]. The interaction of these three systems allows movement, distribution of forces acting on the bony spine, and protection of the spinal cord [4, 5]. The musculoskeletal portion of the spine is part of both the active and passive systems. The passive system consists of the vertebral bodies, the intervertebral discs, ligaments of the spine, the facet joints and the associated joint capsules [4, 5]. The active stabilization system includes the paravertebral muscles, superficial multi-segmental acting muscles including the deeper mono-segmental muscles, and the tendons of these muscles. Thus, the musculature involvement is complex given the required interactions to stabilize the entire spine stable and the individual vertebral segments [4–6]. The neural system has control and feedback functions and thus interacts with the active and passive systems [4, 5].

Imbalances in the stabilization systems can lead to the development of segmental instabilities [7]. In the physiological aging process of striated musculature, there is an increase in fat infiltration (FI) and a decrease in lean musculature (functional cross-sectional area = fCSA) [8, 9]. These two parameters can be determined with MRI muscle measurements. Increased FI and reduced fCSA are taken as surrogate markers for degraded, weaker muscle [10]. The literature highlights that FI is probably the more important parameter in determining muscle functional status. A higher FI is indicative of worse muscle function [11].

Recent studies have shown that increased FI of the posterior paraspinal musculature is associated with spinal pathologies such as degenerative spondylolisthesis, lumbar intervertebral disc degeneration and degenerative lumbar kyphosis [12–15]. It has also been demonstrated that the muscular area (CSA) of the posterior paraspinal muscles (PPM) is associated with the degree of lumbar lordosis. In the case of muscle atrophy or prolonged bed rest that results in weakened muscles, there could be alterations in lumbar lordosis [16–19]. Due to the weakening of the paraspinal muscles from age or degenerative pathologies, the stabilization capacity of the active system might be limited. This can cause segmental instabilities in the sagittal plane and lead to a loss of lumbar lordosis (LL) due to reduced extension capacity. The function of the psoas muscle on the lumbar spine has not been fully elucidated. Whether the psoas muscle has a compensatory function to maintain the stability and lordosis of the lumbar spine is still unknown. The aim of this study was to investigate the interaction between psoas and PPM muscle composition to elucidate if the fat infiltration (FI) of the psoas muscle is associated with the posterior paraspinal morphology.

## Methods

### Subjects

**Fig. 1:**
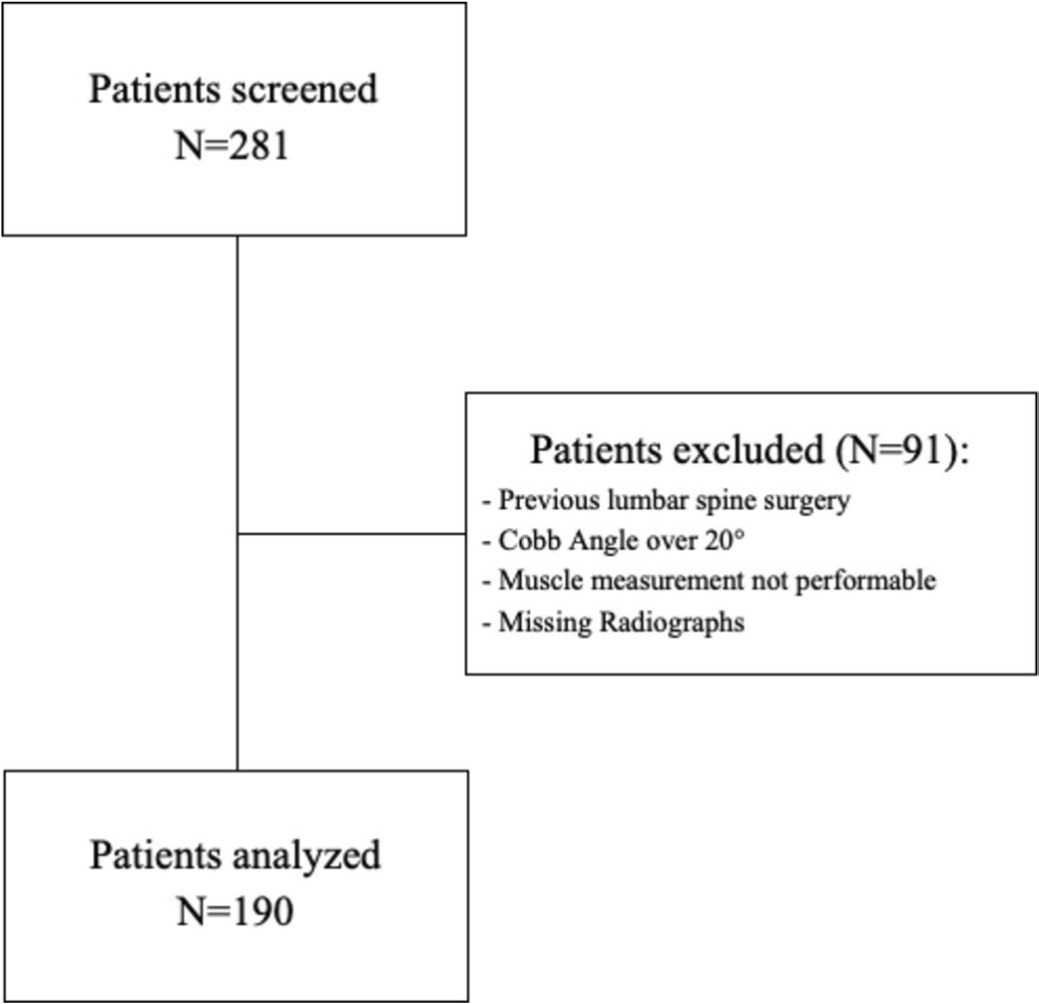
Flowchart of patient inclusion and exclusion

A retrospective review of patients undergoing posterior lumbar fusion between 2014 to 2021 due to degenerative spinal conditions at a single academic institution was conducted. The investigation was approved by the institutional review board and was in compliance with the Helsinki Declaration. The institutional review board at Hospital for Special Surgery waived the requirement for written informed consent due to the retrospective study design. Inclusion criteria included patients > 18 years old, a preoperative magnetic resonance imagining (MRI) of the lumbar spine within 12 months prior to surgery, and availability of a preoperative lumbar radiograph. Exclusion criteria included any previous lumbar spine surgery, a Cobb angle > 20°, missing radiographs and non-measurable MR images due to technical incompatibility (Fig. 1). Patients' records were reviewed for demographic data, diagnoses, treated segments, American Society of Anesthesiology (ASA) score, and common comorbidities.

### Muscle measurements

**Fig. 2:**
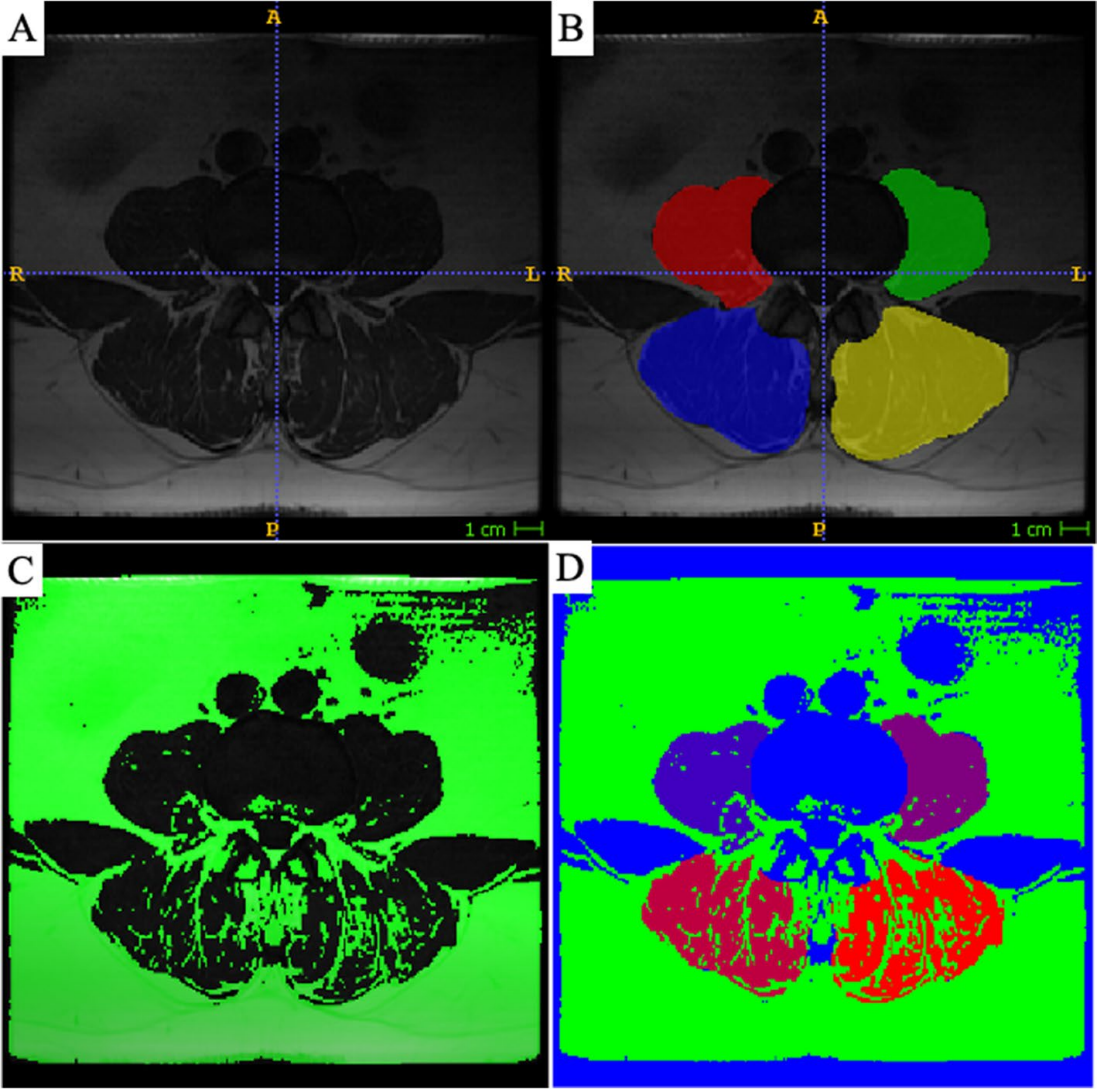
Muscle measurement technique. Images **A** and **B** demonstrate the segmentation process utilizing ITK Snap. Images **C** and **D** highlight the use of the custom written software program and the pixel intensity thresholds

Prior to performing the muscle measurements, the upper endplate of L4 was set as the measurement level because it was previously shown that L4 is predictive for FI of the lumbar paraspinal musculature [20, 21]. The muscles assessed were the psoas muscle and the posterior paraspinal musculature consisting of the erector spinae and multifidus. Using axial T2-weighted MR images, the muscles were segmented utilizing a dedicated software (ITK SNAP version 3.8.0; www.itksnap.org [22];) (Fig. 2A and B). After the segmentation, a custom written software (Matlab version R2019a, The MathWorks, Inc., Natick, MA, USA) was applied to calculate the cross-sectional area (CSA), the functional cross-sectional area (fCSA) and the fat area (FAT) of each muscle (Fig. 2C and D). The calculation is based on pixel intensity thresholds of the segmented muscles and identifies pixels either as fat or muscle by selecting an automatic threshold signal with intensity bias correction by quadratic fitting. Pixels above the threshold are considered as fat and pixels below as muscle. The results of the calculations include the CSA (= fCSA + FAT), fCSA and FAT. Fat infiltration (FI, %) was further calculated for the segmented muscles using the following equation: FI = $\left(\frac{FAT}{CSA}\right)*100$. The right and left sides of the muscles were summarized and normalized by patient height (cm^2^/m^2^). It has recently been demonstrated that the muscle measurement method we used has an excellent intra- and inter-rater reliability [23].

### Lumbar lordosis measurement

Lumbar lordosis (LL) was measured on preoperative lateral lumbar spine radiographs by one orthopaedic resident. LL was determined using the Cobb angle from the superior endplate of L1 and S1.

### Statistical analysis

First, the data were tested for normal distribution using the Shapiro wilk test. Depending on the distribution, mean and standard deviation (SD) or median and interquartile range [IQR] are reported in the manuscript. According to Kjaer et al., patients can be divided into three groups with low (< 10%), medium (10–50%) and high FI (≥ 50%). Since none of our patients had < 10% FI_PPM_, we divided patients into high fat infiltration (≥ 50%) and low to moderate fat infiltration (< 50%) of the PPM [24, 25]. All analyses were stratified by sex. For group comparisons of continuous variables, either the t-test or the Mann–Whitney-U test was conducted depending on the distribution of the data. A multiple linear regression analysis was conducted with FI_Psoas_ as the dependent variable. Age, body mass index, sex, race and FI_PPM_ were the independent variables for the regression model. Spearman rank correlation testing was conducted to determine the associations between the muscular parameters. Statistical significance was set to *p* < 0.05. All analysis were conducted using SPSS Version 28.0 (IBM Corporation, New York, United States).

## Results

A total of 190 patients (57.9% female) with a median age of 64.7 [56.7;71.4] years and median BMI of 28.3 [25.7;32.7] kg/m^2^ were analyzed. The majority of patients were Caucasian (90%) and the most common surgical diagnosis was degenerative spondylolisthesis (81.1%) followed by spinal stenosis (78.9%). 59.4% of patients received a monosegmental posterior fusion and had an ASA score of II (71.6%). A detailed description of patients’ demographics can be found in Table 1 stratified by sex.

**Table 1:** Patient demographics

	**Patient demographics**		
	All	Female	Male
N	190	110	80
Age [years]	64.7 [56.7;71.4]	65.6 [58.3;71.7]	63.1 [54.5;69.9]
BMI [kg/m^2^]	28.3 [25.7;32.7]	27.8 [24.8;32.2]	29.0 [26.9;33.8]
**Race** N (%)			
Caucasian	171 (90)	101 (91.8)	70 (87.5)
African American	12 (6.3)	6 (5.5)	6 (7.5)
Asian	4 (2.1)	1 (0.9)	3 (3.8)
other	3 (1.6)	2 (1.8)	1 (1.2)
Diagnosis* N (%)			
Spinal Stenosis	150 (78.9)	86 (78.2)	64 (80)
Foraminal Stenosis	6 (3.2)	2 (1.8)	4 (5)
DDD	123 (64.7)	62 (56.4)	61 (76.3)
Spondylolisthesis	154 (81.1)	87 (79.1)	67 (83.8)
Neurogenic Claudication	57 (30)	33 (30)	24 (30)
Herniated Nucleus Pulposus	21 (11.1)	13 (11.1)	8 (10)
**Treated Segments** N (%)			
I	113 (59.4)	66 (60)	47 (58.8)
II	63 (33.2)	37 (33.6)	26 (32.5)
III	11 (5.8)	6 (5.5)	5 (6.3)
IV	2 (1.1)	1 (0.9)	1 (1.2)
V	1 (0.5)	0 (0)	1 (1.2)
**ASA-Score** N (%)			
I	8 (4.2)	5 (4.6)	3 (3.8)
II	136 (71.6)	79 (71.8)	57 (71.2)
III	46 (24.2)	26 (23.6)	20 (25.0)
**Comorbidities** N (%)			
Diabetes Mellitus N (%)	17 (8.9)	11 (10.0)	6 (7.5)
COPD N (%)	7 (3.7)	5 (4.6)	2 (2.5)
Hypertension N (%)	77 (40.5)	43 (39.1)	34 (42.5)
Congestive Heart Failure N (%)	0 (0)	0 (0)	0 (0)
Ever Smoker N (%)	78 (41.1)	40 (36.4)	38 (47.5)

Age and body mass index (BMI) is presented as median and interquartile range. Categorical variables are presented as frequencies (= N) and percentage (%). *DDD* Degenerative disc disease, *ASA* *Score* American Society of Anesthesiology Score

### Associations between the posterior paraspinal muscles and the psoas muscle

In females, 65 (59.5%) were found to have FI_PPM_ less than < 50%. In this group of low to medium FI, both FAT_Psoas_ and FI_Psoas_ were significantly lower than in high FI patients with FI_PPM_ ≥ 50%. The majority of males, 82.5% (*n* = 66), had a FI_PPM_ < 50% compared to the 17.5% (*n* = 14) with a FI_PPM_ ≥ 50%. However, A similar picture as in females is seen in males. Men with FI_PPM_ ≥ 50% had a significantly lower FI_Psoas_. Group comparison showed that both FAT_PPM_ and FI_PPM_ were significantly greater in the FI_PPM_ ≥ 50% group. fCSA_PPM_ was significantly lower in the ≥ 50% FI_PPM_ (Table 2).

**Table 2:** Comparison of the female and male groups classified based on fat infiltration of the posterior paraspinal muscles (PPM)

	*Muscle Measurements*				
			< 50% FI_PPM_	≥ 50% FI_PPM_	*p*-value
Females	Psoas	CSA [cm^2^/m^2^]	6.6 [5.6;8.1]	6.7 [6.1;7.8]	0.801
		fCSA [cm^2^/m^2^]	6.0 [5.2;7.3]	6.3 [5.7;7.7]	0.348
		FAT [cm^2^/m^2^]	0.36 [0.16;0.71]	0.23 [0.10;0.43]	**0.016**
		FI [%]	5.6 [2.6;9.7]	3.2 [1.3;6.4]	**0.009**
	PPM	CSA [cm^2^/m^2^]	18.2 [16.2;20.2]	19.9 [17.8;23.6]	**0.002**
		fCSA [cm^2^/m^2^]	11.0 [9.2;12.3]	8.6 [7.4;10.2]	** < 0.001**
		FAT [cm^2^/m^2^]	7.5 [6.2;8.6]	11.4 [9.9;13.5]	** < 0.001**
		FI [%]	40.9 [35.1;46.9]	55.5 [53.4;59.2]	** < 0.001**
Males	Psoas	CSA [cm^2^/m^2^]	9.4 [7.8;11.3]	10.0 [8.1;11.2]	0.685
		fCSA [cm^2^/m^2^]	8.6 [7.4:10.2]	9.6 [8.0;11.0]	0.311
		FAT [cm^2^/m^2^]	0.37 [0.21;0.65]	0.33 [0.10;0.38]	0.068
		FI [%]	3.7 [2.2;7.8]	3.0 [1.0;3.8]	**0.034**
	PPM	CSA [cm^2^/m^2^]	19.5 [17.2;23.0]	17.7 [16.9;19.2]	0.083
		fCSA [cm^2^/m^2^]	12.0 [10.8;14.1]	8.1 [7.1;8.8]	** < 0.001**
		FAT [cm^2^/m^2^]	7.4 [5.8;9.3]	10.3 [9.3;11.0]	** < 0.001**
		FI [%]	38.9 [32.7;44.0]	54.6 [53.1;60.0]	** < 0.001**

Significant values are written in bold. Statistical significance was set at a *p*-value < 0.05. *FI* Fat infiltration; *CSA* Cross sectional area, *fCSA* Functional cross-sectio*nal *area, *FAT* Total fat area

The correlation analysis revealed several significant associations between the posterior paraspinal muscles and the psoas for both men and women. An increase in FI_PPM_ is associated with a significant lower FI_Psoas_ in both men and women (f: *ρ* = -0.403, *p* < 0.001; m: *ρ* = -0.452, *p* < 0.001). Similarly, an increase in FAT_PPM_ leads to a significant decrease in FAT_Psoas_ and FI_Psoas_ in both men and women. However, there was a positive association between fCSA_PPM_ and FI_Psoas_ found only in women. (Table 3).

**Table 3:** Correlation analysis for the muscular parameters stratified for sex

			*Posterior Paraspinal Muscles (PPM)*							
			*Female*				*Male*			
			CSA [cm^2^/m^2^]	fCSA [cm^2^/m^2^]	FAT [cm^2^/m^2^]	FI [%]	CSA [cm^2^/m^2^]	fCSA [cm^2^/m^2^]	FAT [cm^2^/m^2^]	FI [%]
*Psoas*	*Female*	CSA [cm^2^/m^2^]	,337^**^	,366^**^	,136	-,076				
		fCSA [cm^2^/m^2^]	,380^**^	,307^**^	,230^*^	,022				
		FAT [cm^2^/m^2^]	-,076	,299^**^	-,316^**^	-,392^**^				
		FI [%]	-,177	,222^*^	-,383^**^	-,403^**^				
	*Male*	CSA [cm^2^/m^2^]					,370^**^	,350^**^	,239^*^	-,043
		fCSA [cm^2^/m^2^]					,434^**^	,325^**^	,380^**^	,091
		FAT [cm^2^/m^2^]					-,117	,160	-,444^**^	-,427^**^
		FI [%]					-,255^*^	,058	-,558^**^	-,452^**^

Significant values are marked with * or **. *ρ* -values marked with * are significant at the 0.05 level. *ρ* -values marked with ** are significant at the 0.01 level. *CSA* Cross sectional area, *fCSA* Functional cross-sectional area, *FAT* Total f*at area*,* FI* fat infiltration

The overall multiple linear regression model (Table 4) was significant (*p* < 0.001) and represented 17.1% (Corrected R^2^ = 0.171) of the variation of the dependent variable, FI_Psoas_. FI_PPM_ (β = -0.520; *p* < 0.001) and sex (β = 0.151; *p* = 0.032) could predict FI_Psoas_.

**Table 4:** Multiple linear regression model with fat infiltration of the psoas (FI_Psoas_, %) as the dependent variable

Predictor	*b* [*95%-CI*]	*SE*	Beta	*t*	*p*	VIF
(Constant)	11.463 [4.659,18.266]	3.449		3.324	0.001	
FI_PPM_ [%]	-0.297 [-0.391,-0.203]	0.048	-0.520	-6.235	** < 0.001**	1.158
Age [years]	0.070 [-0.012,0.160]	0.041	0.135	1.683	0.094	1.474
BMI [kg/m^2^]	0.024 [-0.112,0.160]	0.069	0.024	0.350	0.727	1.036
Sex	1.870 [0.165,3.576]	0.864	0.151	2.164	**0.032**	1.106
Race	-0.315 [-1.881,1.251]	0.794	-0.027	-0.397	0.692	1.019

Note. R^2^ = .171 (*p* < 0.001) Durbin-Watson Statistics: 1.982

Independent variables were age, body mass index (BMI), sex, race and fat infiltration of the posterior paraspinal muscles (FI_PPM,_ %). Significant values are marked in bold. Statistical sig*nificance was defined as p* < *0.05*

Lumbar lordosis and paraspinal muscle measurements.

There was no significant difference in lumbar lordosis between the groups with < 50% FI_PPM_ and ≥ 50% FI_PPM_ in both males and females (Table 5).

**Table 5:** Lumbar lordosis (LL) in the posterior paraspinal muscle (PPM) groups stratified by sex

	< 50% FI_PPM_	≥ 50% FI_PPM_	*p*-value
Female LL [°]	51.9 ± 13.4	52.3 ± 11.1	0.852
Male LL [°]	49.6 ± 14.8	46.0 ± 9.9	0.392

FI = fat infiltration. Significant values are marked in bold. Statistical sig*nificance was defined as p* < *0.05*

## Discussion

To the authors’ knowledge, this is the first time that a reciprocal relationship between qualitatively assessed FI of the PPM and psoas has been described in males and females. Since FI of the musculature is an important surrogate parameter for muscular function, these results suggest a possible compensatory mechanism of the psoas muscle resulting from increased muscular activity and reduced FI to stabilize the lumbar spine when the PPM are deteriorated.

The stabilization of the lumbar spine is based on three subsystems [4, 5] In addition to the passive and neural systems, the active system is essential for adequate spinal stability. In the absence of the paraspinal muscles, the spine would be highly unstable even under minimal loads [5] It is known that with increasing age, but also with spinal pathologies, the PPM degenerates. Radiologically, this can be measured using surrogate markers such as fCSA and FI where a higher FI implies weaker muscle [10, 11, 26] However, definitive cut off values for the paraspinal muscles have not been established to date to understand when FI is considered pathological.

Özcan-Ekşi et al. have already described a reciprocal relationship between multifidus and psoas in their study for women. Our study confirms the results for women and shows that there is a similar phenomenon for men. In the work presented here, we have the advantage of having qualitative measurements of the musculature, which is particularly advantageous for the psoas muscle, which is known to have little fat infiltration [27].

The function of the PPM on the lumbar spine is of interest to researchers. Due to the high prevalence of back pain and the close functional relationship between the paraspinal musculature and the spine, it is assumed that the paraspinal muscular morphology, especially a higher FI and lower fCSA, is associated with the development of back pain [28–31]. However, the literature is still inconclusive. Further studies investigated the relationship between degenerative spinal diseases and the paraspinal musculature [32–34]. A biomechanical study showed that weakened PPM are associated with the development and worsening of degenerative spondylolisthesis [35]. Clinical studies have shown that the fCSA was smaller and the ratio of fCSA to CSA was higher in patients without degenerative spondylolisthesis [13]. Another study demonstrated that degenerative lumbar kyphosis was associated with a significantly lower fCSA and higher FI of the PPM [15].

In recent years, research has indicated the importance of the musculature is for upright posture [36, 37]. However, the relationship between LL and PPM has not been fully elucidated. Biomechanical studies assume that the PPM generates a follower load in the lumbar spine [38]. The concept of a follower load is based on the fact that the resulting force of the PPM is tangential to the sagittal spinal curve to support the lumbar spine in carrying the weight of the upper body [6, 39]. Increased LL requires a higher follower load and thus stronger PPM [40]. Studies have shown that there is a relationship between CSA_PPM_ and LL, but it has not been shown if this relationship is causative or correlative [38].

Some studies assume that the strength of the muscles is proportional to their CSA [38, 41, 42]. However, this concept is questionable in the PPM since longitudinal studies have shown that there is no change in the CSA at L3/4 of the PPM with age, but a shift to higher FI in patients at the age of 50 years at baseline [43]. Whether age-related loss of LL is due to weakened PPM muscles or due to degenerative changes has not been determined. It is also possible that LL changes only as an adaptation mechanism to changes in pelvic tilt [44]. This concept has recently been proposed but has not been validated by longitudinal studies. The currently accepted concept is that the loss of LL is due to degenerative changes in the intervertebral disc [1]. However, our data reveals no significant differences for both sexes regarding the LL between < 50% FI_PPM_ and ≥ 50% FI_PPM_, which may indicate that LL may be more tightly regulated by other factors than the PPM such as intervertebral disc degenerations, increased pelvic tilt or anterior wedging of the vertebrae.

The function of the psoas muscle on the lumbar spinal column remains unclear. Three main theories about psoas muscle function have been proposed: 1) reduction of lumbar lordosis by bending the trunk forward, 2) increase in LL, and 3) stabilization of the lordotic curve by adaptation of the contraction of the individual fascicles [45–54]. Our data suggest that the psoas may help maintain LL even if the PPM has a higher FI, which, is in line with the third proposed theory. The significantly lower FI_Psoas_ in both men and women with ≥ 50% FI_PPM_ indicates there may be increased psoas muscular activity resulting in a lower FI of the muscle. The potential higher activity in the psoas muscle may be necessary to maintain LL to allow optimal force distribution across the lumbar spine.

Arbanas et al. demonstrated in their study that patients with low back pain (LBP) have a significantly larger CSA_Psoas_ than patients without LBP [55]. However, the FI in the work of Arbanas et al. was only measured quantitatively based on a four grade visual scale. Due to the relatively low FI of the psoas, they were probably unable to demonstrate any significant differences for the FI_Psoas_. Arbans et al. hypothesized that the psoas may compensate by increasing activity to maintain the stability of the lumbar spine as the CSA_Psoas_ was larger in the group with LBP [55].

Our study is not free of limitations. First, causality cannot be established due to our retrospective cross-sectional study design. Additionally due to the retrospective design, we were unable to include other factors influencing muscle composition such as physical activity in our study. Well-designed prospective longitudinal studies are necessary to control for co-factors such as physical activity and to establish causality. Furthermore, it must be noted that only 14 males with a FI_PPM_ ≥ 50% were in our patient population and therefore the results for males must be interpreted with caution. Another point that needs to be addressed is that only patients undergoing lumbar fusion surgery due to degenerative spinal pathologies were included, which limits the generalizability of our results. However, our study population represents a cohort that is frequently seen in orthopedic practice and therefore of high clinical relevance. Another limitation of our study is the uncertainty and variation in posture affecting the LL, and the possibility of changes of the LL throughout the day. However, due to the cross-sectional study design, it is not possible to observe this exactly. We think that due to the relatively large number of patients, the statement of the LL in our study is valid.

## Conclusion

In conclusion, our work provides indications that the psoas might have a compensatory function in stabilizing the lumbar spine and maintaining lumbar lordosis when the posterior paraspinal muscles are degenerated. However, further studies are needed to verify our findings and hypothesis that the psoas muscle is more active when the posterior paraspinal muscles are deteriorated.

## Data Availability

The datasets generated and analyzed during the current study are available from the corresponding author on reasonable request.

## References

[1] Le Huec JC, Roussouly P (2011). Sagittal spino-pelvic balance is a crucial analysis for normal and degenerative spine. Eur Spine J.

[2] Le Huec JC, Saddiki R, Franke J, Rigal J, Aunoble S (2011). Equilibrium of the human body and the gravity line: the basics. Eur Spine J.

[3] Niemitz C (2010). The evolution of the upright posture and gait–a review and a new synthesis. Naturwissenschaften.

[4] Panjabi MM (1992). The stabilizing system of the spine. Part I. Function, dysfunction, adaptation, and enhancement. J Spinal Disord..

[5] Izzo R, Guarnieri G, Guglielmi G, Muto M (2013). Biomechanics of the spine. Part I: spinal stability. Eur J Radiol..

[6] Kim K, Kim YH (2008). Role of trunk muscles in generating follower load in the lumbar spine of neutral standing posture. J Biomech Eng.

[7] Quint U, Wilke HJ, Shirazi-Adl A, Parnianpour M, Loer F, Claes LE (1998). Importance of the intersegmental trunk muscles for the stability of the lumbar spine. A biomechanical study in vitro. Spine (Phila Pa 1976).

[8] Marcus RL, Addison O, Kidde JP, Dibble LE, Lastayo PC (2010). Skeletal muscle fat infiltration: impact of age, inactivity, and exercise. J Nutr Health Aging.

[9] Hamrick MW, McGee-Lawrence ME, Frechette DM (2016). Fatty infiltration of skeletal muscle: Mechanisms and comparisons with bone marrow adiposity. Front Endocrinol (Lausanne).

[10] Visser M, Kritchevsky SB, Goodpaster BH, Newman AB, Nevitt M, Stamm E (2002). Leg muscle mass and composition in relation to lower extremity performance in men and women aged 70 to 79: the health, aging and body composition study. J Am Geriatr Soc.

[11] Goodpaster BH, Park SW, Harris TB, Kritchevsky SB, Nevitt M, Schwartz AV (2006). The loss of skeletal muscle strength, mass, and quality in older adults: the health, aging and body composition study. J Gerontol A Biol Sci Med Sci.

[12] Shi L, Yan B, Jiao Y, Chen Z, Zheng Y, Lin Y (2022). Correlation between the fatty infiltration of paraspinal muscles and disc degeneration and the underlying mechanism. BMC Musculoskelet Disord.

[13] Lee ET, Lee SA, Soh Y, Yoo MC, Lee JH, Chon J. Association of Lumbar Paraspinal Muscle Morphometry with Degenerative Spondylolisthesis. Int J Environ Res Public Health. 2021;18(8):4037. 10.3390/ijerph18084037. 10.3390/ijerph18084037 PMC8070567 33921317

[14] Thakar S, Sivaraju L, Aryan S, Mohan D, Sai Kiran NA, Hegde AS (2016). Lumbar paraspinal muscle morphometry and its correlations with demographic and radiological factors in adult isthmic spondylolisthesis: a retrospective review of 120 surgically managed cases. J Neurosurg Spine.

[15] Kang CH, Shin MJ, Kim SM, Lee SH, Lee CS (2007). MRI of paraspinal muscles in lumbar degenerative kyphosis patients and control patients with chronic low back pain. Clin Radiol.

[16] Sinaki M, Itoi E, Rogers JW, Bergstralh EJ, Wahner HW (1996). Correlation of back extensor strength with thoracic kyphosis and lumbar lordosis in estrogen-deficient women. Am J Phys Med Rehabil.

[17] Belavy DL, Armbrecht G, Richardson CA, Felsenberg D, Hides JA (2011). Muscle atrophy and changes in spinal morphology: is the lumbar spine vulnerable after prolonged bed-rest?. Spine (Phila Pa 1976).

[18] De Martino E, Hides J, Elliott JM, Hoggarth M, Zange J, Lindsay K (2021). Lumbar muscle atrophy and increased relative intramuscular lipid concentration are not mitigated by daily artificial gravity after 60-day head-down tilt bed rest. J Appl Physiol (1985).

[19] Meakin JR, Fulford J, Seymour R, Welsman JR, Knapp KM (2013). The relationship between sagittal curvature and extensor muscle volume in the lumbar spine. J Anat.

[20] Crawford RJ, Filli L, Elliott JM, Nanz D, Fischer MA, Marcon M (2016). Age- and level-dependence of fatty infiltration in lumbar paravertebral muscles of healthy volunteers. AJNR Am J Neuroradiol.

[21] Faron A, Luetkens JA, Schmeel FC, Kuetting DLR, Thomas D, Sprinkart AM (2019). Quantification of fat and skeletal muscle tissue at abdominal computed tomography: associations between single-slice measurements and total compartment volumes. Abdom Radiol (NY).

[22] Yushkevich PA, Piven J, Hazlett HC, Smith RG, Ho S, Gee JC (2006). User-guided 3D active contour segmentation of anatomical structures: significantly improved efficiency and reliability. Neuroimage.

[23] Moser M, Adl Amini D, Jones C, Zhu J, Okano I, et al. The predictive value of psoas and paraspinal muscle parameters measured on MRI for severe cage subsidence after standalone lateral lumbar interbody fusion. Spine J. 2023;23(1):42-53. 10.1016/j.spinee.2022.03.009. Epub 2022 Mar 26. 10.1016/j.spinee.2022.03.009 35351664

[24] Kjaer P, Bendix T, Sorensen JS, Korsholm L, Leboeuf-Yde C (2007). Are MRI-defined fat infiltrations in the multifidus muscles associated with low back pain?. BMC Med.

[25] Kalichman L, Klindukhov A, Li L, Linov L (2016). Indices of paraspinal muscles degeneration: reliability and association with facet joint osteoarthritis: feasibility study. Clin Spine Surg.

[26] Hilton TN, Tuttle LJ, Bohnert KL, Mueller MJ, Sinacore DR (2008). Excessive adipose tissue infiltration in skeletal muscle in individuals with obesity, diabetes mellitus, and peripheral neuropathy: association with performance and function. Phys Ther.

[27] Ozcan-Eksi EE, Eksi MS, Turgut VU, Canbolat C, Pamir MN (2021). Reciprocal relationship between multifidus and psoas at L4–L5 level in women with low back pain. Br J Neurosurg.

[28] Wu A, March L, Zheng X, Huang J, Wang X, Zhao J (2020). Global low back pain prevalence and years lived with disability from 1990 to 2017: estimates from the Global Burden of Disease Study 2017. Ann Transl Med.

[29] Kalichman L, Carmeli E, Been E (2017). The association between imaging parameters of the paraspinal muscles, spinal degeneration, and low back pain. Biomed Res Int.

[30] Teichtahl AJ, Urquhart DM, Wang Y, Wluka AE, Wijethilake P, O'Sullivan R (2015). Fat infiltration of paraspinal muscles is associated with low back pain, disability, and structural abnormalities in community-based adults. Spine J.

[31] Ranger TA, Cicuttini FM, Jensen TS, Peiris WL, Hussain SM, Fairley J (2017). Are the size and composition of the paraspinal muscles associated with low back pain? A systematic review. Spine J.

[32] Kalichman L, Hodges P, Li L, Guermazi A, Hunter DJ (2010). Changes in paraspinal muscles and their association with low back pain and spinal degeneration: CT study. Eur Spine J.

[33] Abbas J, Slon V, May H, Peled N, Hershkovitz I, Hamoud K (2016). Paraspinal muscles density: a marker for degenerative lumbar spinal stenosis?. BMC Musculoskelet Disord.

[34] Ding JZ, Kong C, Li XY, Sun XY, Lu SB, Zhao GG (2022). Different degeneration patterns of paraspinal muscles in degenerative lumbar diseases: a MRI analysis of 154 patients. Eur Spine J.

[35] Zhu R, Niu WX, Zeng ZL, Tong JH, Zhen ZW, Zhou S (2017). The effects of muscle weakness on degenerative spondylolisthesis: A finite element study. Clin Biomech (Bristol, Avon).

[36] Hori Y, Hoshino M, Inage K, Miyagi M, Takahashi S, Ohyama S (2019). ISSLS prize in clinical science 2019: clinical importance of trunk muscle mass for low back pain, spinal balance, and quality of life-a multicenter cross-sectional study. Eur Spine J.

[37] Muellner M, Haffer H, Chiapparelli E, Dodo Y, Tan ET, Shue J, et al. Differences in lumbar paraspinal muscle morphology in patients with sagittal malalignment undergoing posterior lumbar fusion surgery. Eur Spine J. 2022;31(11):3109–18. 10.1007/s00586-022-07351-3. Epub 2022 Aug 29. 10.1007/s00586-022-07351-3 PMC10585706 36038784

[38] Sparrey CJ, Bailey JF, Safaee M, Clark AJ, Lafage V, Schwab F (2014). Etiology of lumbar lordosis and its pathophysiology: a review of the evolution of lumbar lordosis, and the mechanics and biology of lumbar degeneration. Neurosurg Focus.

[39] Patwardhan AG, Havey RM, Meade KP, Lee B, Dunlap B (1999). A follower load increases the load-carrying capacity of the lumbar spine in compression. Spine (Phila Pa 1976)..

[40] Meakin JR, Aspden RM (2012). Modeling the effect of variation in sagittal curvature on the force required to produce a follower load in the lumbar spine. J Mechan Med Biol.

[41] Lee HJ, Lim WH, Park JW, Kwon BS, Ryu KH, Lee JH (2012). The relationship between cross sectional area and strength of back muscles in patients with chronic low back pain. Ann Rehabil Med.

[42] Maughan RJ, Watson JS, Weir J (1983). Strength and cross-sectional area of human skeletal muscle. J Physiol.

[43] Fortin M, Videman T, Gibbons LE, Battie MC (2014). Paraspinal muscle morphology and composition: a 15-yr longitudinal magnetic resonance imaging study. Med Sci Sports Exerc.

[44] Muellner M, Haffer H, Moser M, Chiapparelli E, Dodo Y, Adl Amini D, et al. Paraspinal musculature impairment is associated with spinopelvic and spinal malalignment in patients undergoing lumbar fusion surgery. Spine J. 2022;22(12):2006–16. 10.1016/j.spinee.2022.07.103. Epub 2022 Aug 6. 10.1016/j.spinee.2022.07.103 PMC10623672 35944826

[45] Penning L (2000). Psoas muscle and lumbar spine stability: a concept uniting existing controversies. Critical review and hypothesis. Eur Spine J..

[46] Santaguida PL, McGill SM (1995). The psoas major muscle: a three-dimensional geometric study. J Biomech.

[47] Hansen L, de Zee M, Rasmussen J, Andersen TB, Wong C, Simonsen EB (2006). Anatomy and biomechanics of the back muscles in the lumbar spine with reference to biomechanical modeling. Spine (Phila Pa 1976).

[48] Regev GJ, Kim CW, Tomiya A, Lee YP, Ghofrani H, Garfin SR (2011). Psoas muscle architectural design, in vivo sarcomere length range, and passive tensile properties support its role as a lumbar spine stabilizer. Spine (Phila Pa 1976).

[49] Andersson E, Oddsson L, Grundstrom H, Thorstensson A (1995). The role of the psoas and iliacus muscles for stability and movement of the lumbar spine, pelvis and hip. Scand J Med Sci Sports.

[50] Park RJ, Tsao H, Claus A, Cresswell AG, Hodges PW (2013). Changes in regional activity of the psoas major and quadratus lumborum with voluntary trunk and hip tasks and different spinal curvatures in sitting. J Orthop Sports Phys Ther.

[51] Jorgensen MJ, Marras WS, Granata KP, Wiand JW (2001). MRI-derived moment-arms of the female and male spine loading muscles. Clin Biomech (Bristol, Avon).

[52] Jorgensson A (1993). The iliopsoas muscle and the lumbar spine. Aust J Physiother.

[53] Bogduk N, Pearcy M, Hadfield G (1992). Anatomy and biomechanics of psoas major. Clin Biomech (Bristol, Avon).

[54] Nachemson A (1966). Electromyographic studies on the vertebral portion of the psoas muscle; with special reference to its stabilizing function of the lumbar spine. Acta Orthop Scand.

[55] Arbanas J, Pavlovic I, Marijancic V, Vlahovic H, Starcevic-Klasan G, Peharec S (2013). MRI features of the psoas major muscle in patients with low back pain. Eur Spine J.

